# Tumor Testing and Genetic Analysis to Identify Lynch Syndrome Patients in an Italian Colorectal Cancer Cohort

**DOI:** 10.3390/cancers15205061

**Published:** 2023-10-19

**Authors:** Antonino Pantaleo, Giovanna Forte, Filomena Cariola, Anna Maria Valentini, Candida Fasano, Paola Sanese, Valentina Grossi, Antonia Lucia Buonadonna, Katia De Marco, Martina Lepore Signorile, Anna Filomena Guglielmi, Andrea Manghisi, Gianluigi Gigante, Raffaele Armentano, Vittoria Disciglio, Cristiano Simone

**Affiliations:** 1 Medical Genetics, National Institute of Gastroenterology-IRCCS “Saverio de Bellis” Research Hospital, Castellana Grotte, 70013 Bari, Italy; antonino.pantaleo@irccsdebellis.it (A.P.); forte.labsimone@gmail.com (G.F.); filo.cariola@irccsdebellis.it (F.C.); fasano.labsimone@gmail.com (C.F.); sanese.labsimone@gmail.com (P.S.); grossi.labsimone@gmail.com (V.G.); lucia.buonadonna@irccsdebellis.it (A.L.B.); demarco.labsimone@gmail.com (K.D.M.); leporesignorile.labsimone@gmail.com (M.L.S.); floranna.guglielmi@irccsdebellis.it (A.F.G.); andrea.manghisi@irccsdebellis.it (A.M.); 2 Department of Pathology, National Institute of Gastroenterology-IRCCS “Saverio de Bellis” Research Hospital, Castellana Grotte, 70013 Bari, Italy; am.valentini@irccsdebellis.it (A.M.V.); raffaele.armentano@irccsdebellis.it (R.A.); 3 Department of General Surgery, National Institute of Gastroenterology-IRCCS “Saverio de Bellis” Research Hospital, Castellana Grotte, 70013 Bari, Italy; gianluigi.gigante@gmail.com; 4 Medical Genetics, Department of Precision and Regenerative Medicine and Jonic Area (DiMePRe-J), University of Bari Aldo Moro, 70124 Bari, Italy

**Keywords:** Lynch syndrome, colorectal cancer, mismatch repair (MMR) genes, variant of unknown significance (VUS), tailored genetic counseling

## Abstract

**Simple Summary:**

Lynch syndrome (LS) is an inherited genetic condition caused by germline mutations in DNA mismatch repair (MMR) genes. It is associated with a predisposition to different types of cancer, including colorectal cancer (CRC). CRC is the fourth most common cancer worldwide. The screening algorithm for the selection of LS patients is based on the identification of CRC specimens that have MMR loss/high microsatellite instability (MSI-H) and are wild-type for *BRAF*^V600^. The aim of this retrospective study was to clinically and molecularly characterize CRC patients with these features. We used a comprehensive approach including tumor testing for the assessment of MSI status, clinical evaluation of patients and their families, and genetic analysis to identify variants in MMR and other cancer-related genes. The clinical and molecular characterization of these patients highlights the importance of personalized medicine to provide tailored genetic counseling, management, and surveillance to families with LS and hereditary cancer.

**Abstract:**

Lynch syndrome (LS) is an inherited cancer susceptibility syndrome caused by germline mutations in a DNA mismatch repair (MMR) gene or in the *EPCAM* gene. LS is associated with an increased lifetime risk of colorectal cancer (CRC) and other malignancies. The screening algorithm for LS patient selection is based on the identification of CRC specimens that have MMR loss/high microsatellite instability (MSI-H) and are wild-type for *BRAF*^V600^. Here, we sought to clinically and molecularly characterize patients with these features. From 2017 to 2023, 841 CRC patients were evaluated for MSI and *BRAF*^V600E^ mutation status, 100 of which showed MSI-H. Of these, 70 were wild-type for *BRAF*^V600^. Among these 70 patients, 30 were genetically tested for germline variants in hereditary cancer predisposition syndrome genes. This analysis showed that 19 of these 30 patients (63.3%) harbored a germline pathogenic or likely pathogenic variant in MMR genes, 2 (6.7%) harbored a variant of unknown significance (VUS) in MMR genes, 3 (10%) harbored a VUS in other cancer-related genes, and 6 (20%) were negative to genetic testing. These findings highlight the importance of personalized medicine for tailored genetic counseling, management, and surveillance of families with LS and other hereditary cancer syndromes.

## 1. Introduction

Colorectal cancer (CRC) is the fourth most common cancer worldwide, ranking third in cancer mortality [1]. Several factors can predispose to the development of CRC. These include modifiable (e.g., smoking, physical inactivity, and alcohol) and nonmodifiable risk factors (e.g., hereditary tumor syndromes and inflammatory bowel disease) [2]. The most common hereditary syndrome related to CRC is Lynch syndrome (LS), also known as hereditary nonpolyposis colorectal cancer (HNPCC), which accounts for 2–3% of all CRCs [3]. LS patients have an earlier average age of CRC onset and a lifetime cumulative CRC risk of up to 52.2% in women and 68.7% in men [4].

Predisposition to LS is associated with heterozygous germline pathogenic alterations in the DNA mismatch repair genes *MLH1*, *MSH2*, *MSH6*, and *PMS2*. Moreover, germline deletions affecting the *EPCAM* gene and leading to transcriptional silencing of the *MSH2* gene have also been identified in LS patients. Patients carrying germline pathogenic variants in one of these genes have an increased risk of developing extracolonic malignancies, including endometrial, pancreatic, gastric, ovarian, ureter/renal, biliary tract, prostate, brain, and small intestinal cancers. LS-related CRCs show genomic alterations that are considered LS hallmarks. Deficiency of the mismatch repair (MMR) system causes the accumulation of insertions/deletions in short tandem repeats, which are also known as microsatellites. This replication and repair error phenotype is called microsatellite instability (MSI) [5,6]. In LS, CRCs exhibit a high MSI profile (MSI-H) [7]. According to European guidelines, all CRCs should be tested by MMR (*MLH1*, *MSH2*, *MSH6*, and *PMS2*) immunohistochemistry or MSI analysis to screen for LS [8]. The assessment of the MSI status is a sensitive but not very specific method, as most MSI-H CRCs are sporadic and, therefore, not related to LS [9]. However, other CRC genotypic features can guide the diagnostic process. The *BRAF*^V600E^ somatic variant is one of them. Indeed, it is considered a strong negative predictor of LS [10]. Moreover, MSI testing and analysis of the *BRAF*^V600E^ somatic variant are also emerging as clinically relevant tools for prognosis and for the evaluation of the best therapeutic strategy in CRC patients [11]. As a result, these tests are now performed routinely. In the present retrospective study, we report our six-year experience evaluating CRC patients with LS-related phenotype. In particular, we contextualize the genotypic findings of these probands based on their clinical picture and family history.

## 2. Materials and Methods

### 2.1. Patient Recruitment

Between January 2017 and July 2023, a total of 841 continuous patients who underwent CRC surgery at the National Institute of Gastroenterology “Saverio de Bellis”, Castellana Grotte, Bari, Italy, were included in our study. All 841 patients with CRC were involved in this study without inclusion and exclusion criteria. Written informed consents to perform molecular testing and further studies on blood and tissue specimens were obtained from the patients and their relatives using a form approved by the competent ethics committee, in line with the principles of the Declaration of Helsinki and any other applicable local ethical and legal requirement (protocol code N_170, date of approval 31 October 2016). All CRC tissue specimens collected from these 841 patients were subjected to molecular testing to evaluate the MSI status. Moreover, all patients provided blood samples for prospective genetic testing. Clinicopathological and demographic information was recorded for each patient. 

### 2.2. Microsatellite Instability Assay and BRAF Mutation Assay

Detection of the MSI status was performed on formalin-fixed paraffin-embedded (FFPE) CRC specimens from the above-mentioned 841 patients using the fully automated real-time PCR system Idylla MSI Test (Biocartis, Mechel, Belgium) according to the manufacturer’s protocol. Briefly, the assay was performed on 5–10 µm FFPE CRC tissue sections to analyze seven monomorphic biomarkers located on seven tumor-specific genes (*ACVR2A*, *BTBD7*, *DIDO1*, *MRE11*, *RYR3*, *SEC31A*, and *SULF2*). Detection of the *BRAF*^V600E^ substitution was performed using the Idylla *BRAF* Mutation Test (Biocartis) according to the manufacturer’s protocol. Briefly, 5–10 µm FFPE CRC tissue sections were mounted on disposable cartridges to perform allele-specific PCR reactions for the identification of wild-type (WT) or V600E-mutated *BRAF*. Before carrying out the MSI and *BRAF*^V600E^ tests, the tumor content of tissue samples was determined by estimating the percentage of neoplastic cells on hematoxylin- and eosin-stained whole slides, and, if appropriate, macrodissection was performed to achieve a tumor cell content of at least 50%. 

### 2.3. DNA Extraction and Copy Number Analysis

Genomic DNA was extracted from peripheral blood with the QIAamp DNA Blood Mini Kit (Qiagen, Hilden, Germany) according to the manufacturer’s instructions. To examine putative germline pathogenic copy number variants (CNVs) affecting MMR genes (*MLH1*, *MSH2*, *MSH6*, and *PMS2*) and *EPCAM*, CNV analysis was performed on whole-blood-extracted DNA by multiplex ligation-dependent probe amplification (MLPA) using the SALSA MLPA P003-D1 *MLH1/MSH2*, SALSA MLPA P072-D1 *MSH6/MUTYH*, and SALSA MLPA P008-D1 *PMS2* kits (MRC-Holland, Amsterdam, The Netherlands) according to the manufacturer’s instructions. The amplification products were separated on an ABI Prism 3130 Genetic Analyser (Thermo Fisher Scientific, Waltham, MA, USA). Migration of fragments was calculated by comparison to the GeneScan LIZ-500 size standard (Thermo Fisher Scientific). Data analysis was performed using Coffalyser software v.220513.1739 (MRC Holland).

### 2.4. Sanger Sequencing and Next-Generation Sequencing

Patients with MSI-H and *BRAF*^V600^ WT CRC underwent genetic counseling and were genetically tested. In particular, patients meeting classical Bethesda and/or Amsterdam criteria for LS were analyzed by Sanger sequencing and copy number variation detection for MMR genes, whereas patients without an LS-related cancer family history and/or with a family history of cancers potentially associated with other major hereditary tumor predisposition syndromes were analyzed by next-generation sequencing (NGS) and copy number variation detection for MMR genes. The complete coding region of the *MLH1*, *MSH2*, *MSH6*, and *PMS2* genes was screened for mutations using primer sequences previously published by Holinski et al., Kolodner et al., and Vaughn et al. [12,13,14]. Briefly, PCR sequencing and capillary electrophoresis were performed on an Applied Biosystems 3130 Genetic Analyzer (Thermo Fisher Scientific). Mutations and polymorphisms were confirmed in independently amplified PCR products. NGS was performed using the Ion AmpliSeq Custom Panel (BRCA Reflex), which enables the analysis of 25 genes involved in major hereditary cancer predisposition syndromes (*APC*, *ATM*, *BARD1*, *BMPR1A*, *BRIP1*, *CDH1*, *CDK4*, *CDKN2A*, *CHEK2*, *EPCAM*, *MLH1*, *MRE11A*, *MSH2*, *MSH6*, *MUTYH*, *NBN*, *PALB2*, *PMS2*, *PTEN*, *RAD50*, *RAD51C*, *RAD51D*, *SMAD4*, *STK11*, and *TP53*). Briefly, 10 ng of whole-blood-extracted DNA was used to generate libraries using the Ion AmpliSeq Chef Solutions DL8 Kit (Thermo Fisher Scientific) on an Ion Chef System (Thermo Fisher Scientific). Subsequently, the prepared libraries were sequenced on an Ion GeneStudio S5 Prime System (Thermo Fisher Scientific) using the Ion 510™ & Ion 520™ & Ion 530™ Kit and the Ion 520 Chip Kit (Thermo Fisher Scientific) according to the manufacturer’s instructions. Data analysis was performed using Torrent Suite Software v.5.12.1 (Thermo Fisher Scientific). Reads were aligned to the hg19 human reference genome. The mean average read depth and the percentage of reads mapping to the region of interest (ROI) out of the total number of reads (reads on target) were calculated using the Coverage Analysis plugin (Torrent Suite v.5.12.1 software, Thermo Fisher Scientific). For each sample, the ROI percentage with a minimum coverage of 20X was calculated using the amplicon coverage matrix file. The genetic variants identified were confirmed by Sanger sequencing analysis. Primer sequences are available upon request.

### 2.5. Variant Classification

The clinical significance of each variant was annotated using the American College of Medical Genetics and Genomics/Association for Molecular Pathology (ACMG/AMP) guidelines [15] as well as the pathogenicity assertions registered in ClinVar.

### 2.6. In Silico Pathogenicity Prediction Analysis

Three in silico tools were used to predict the pathogenicity of the missense mutations identified in the protein variants under study. Panther db (**P**rotein **AN**alysis **TH**rough **E**volutionary **R**elationships; https://www.pantherdb.org/ (accessed on 20 July 2023)) is a publicly accessible resource reporting protein evolution and function information represented by phylogenetic trees [16,17]. It offers software tools for protein sequence analysis tasks, such as investigating functional aspects of genes, performing enrichment analysis and homology annotation, and evaluating the impact of genetic variants at specific protein sites. PolyPhen-2 (Polymorphism Phenotyping v2; http://genetics.bwh.harvard.edu/pph2/ (accessed on 20 July 2023), a method based on the Nave Bayes Classifier, incorporates sequence- and structure-based characteristics [18]. Sorting Intolerant From Tolerant (SIFT) is a position-specific scoring matrix-based algorithm that predicts the deleterious nature of a mutation based on sequence homology using the Position-Specific Iterated BLAST (PSI-BLAST) method [19]. In particular, SIFT predicts deleterious changes in conserved protein regions by considering the position and type of the amino acid change. All in silico prediction analyses were performed using the default parameter settings of the tools.

## 3. Results

### 3.1. Genetic Testing of CRC Patients for LS

**Figure 1:**
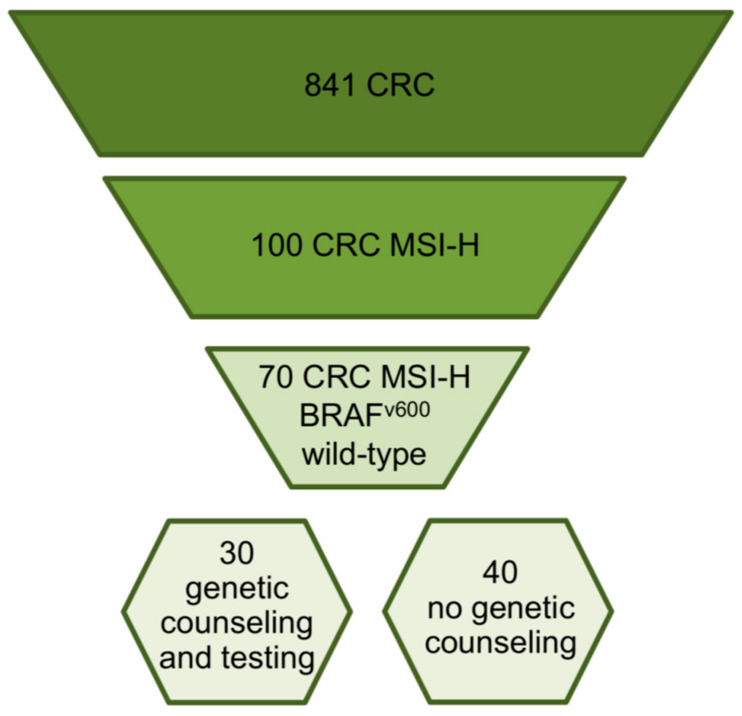
Flow chart describing the study design. Colorectal (CRC) specimens (n = 841) collected from January 2017 to July 2023 were analyzed for their microsatellite instability (MSI) status. Next, MSI-high (MSI-H) specimens (n = 100) were analyzed for *BRAF*^V600^ status. Of these, 70/100 CRC specimens were wild-type (WT) for MSI-H and *BRAF*^V600^. Patients with MSI-H and *BRAF*^V600^ WT CRC (n = 30) were genetically tested for germline variants in genes associated with major hereditary cancer predisposition syndromes. The remaining patients with MSI-H and *BRAF*^V600^ WT CRC (n = 40) did not undergo genetic counseling.

Between January 2017 and July 2023, a total of 841 patients underwent CRC surgery at the National Institute of Gastroenterology “Saverio de Bellis”. All 841 patients with CRC were involved in this study without inclusion and exclusion criteria. CRC specimens were obtained from these patients and analyzed for their MSI status. Of these, 100 were found positive, i.e., MSI-H, and were subjected to further molecular characterization for the *BRAF*^V600E^ substitution. This analysis identified the *BRAF*^V600E^ variant in 30 specimens (30%), while the remaining 70 (70%) were WT for *BRAF*^V600^ (Figure 1).

Genetic counseling was suggested for the 70 patients with MSI-H and *BRAF* WT CRC. Of these, only 30 (42.9%) agreed to do so and were genetically tested for germline variants in genes associated with LS and/or other major hereditary cancer predisposition syndromes. The remaining 40 patients did not undergo genetic counseling at our Institute (Figure 1). During genetic counseling, a detailed personal and family history of CRC, endometrial cancer (EC), and extracolonic malignancies was obtained for all 30 suspected LS patients and their families. Evaluation of this information supported the decision to perform genetic testing on these patients. Specifically, 19 suspected LS patients with a strong personal and/or family LS-related cancer history meeting the Amsterdam or Bethesda criteria were analyzed to evaluate genetic alterations in MMR genes (*MLH1*, *MSH2*, *MSH6*, and *PMS2*) and the *EPCAM* gene, while the remaining 11 suspected LS patients, who had a personal but not a family cancer history and/or had a family history of cancers potentially correlated to other major hereditary tumor predisposition syndromes, were subjected to genetic analysis of 25 genes involved in major hereditary cancer predisposition syndromes by NGS. The clinicopathological features of these 30 CRC patients (16 females and 14 males) are summarized in Table 1 and Table S1.

**Figure 2:**
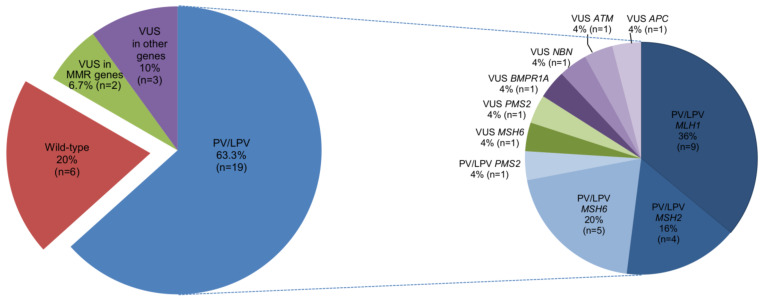
Percentage distribution of gene variants in patients subjected to genetic testing for germline variants in hereditary cancer predisposition syndrome genes and with high microsatellite instability and *BRAF*^V600^ wild-type CRC. Abbreviations: CRC: colorectal cancer; LPV: likely pathogenic variant; MMR: mismatch repair; PV: pathogenic variant; VUS: variant of unknown significance.

Eight patients were diagnosed with CRC before and 22 at or after the age of 50 years. Twenty-nine patients had a single CRC and one patient presented with multiple CRCs. Among LS-related malignancies, EC was the most frequently observed cancer (eight patients, 26.7%). As regards family history, 29/30 patients (96.7%) had a family history of cancer. Concerning the results of the genetic testing for LS, 19 patients (63.3%) had a germline pathogenic (PV) or likely pathogenic variant (LPV) in a MMR gene (*MLH1:* c.380+2T>C; c.545+3A>G; c.731G>A; c.1961C>T, *MSH2*: c.943-1G>A; c.1681G>T; c.1786_1788delAAT; c.2635-2A>G, *MSH6*: c.1957_1960GTGAdup; c.-150_426del, *PMS2:* c.1987G>T), 2 (6.7%) had a variant of unknown significance (VUS) in a MMR gene (*MSH2*, *MSH6*: c.663A>C, *PMS2:* c.184G>A), 3 (10%) had a VUS in genes (*ATM:* c.3563A>C, *NBN:* c.839C>T, *APC*: c.2780C>G, and *BMPR1A:* c.1498A>G) related to other major cancer predisposition syndromes, and 6 (20%) were WT for all the analyzed genes (Table S2, Figure 2).

### 3.2. Patients with Germline Genetic Variants in MMR Genes

The presence of MMR genetic alterations was assessed in the blood samples collected from the 30 suspected LS patients. In total, 16 out of 19 patients (84.2%) were identified as harboring PVs and/or LPVs in an MMR gene by performing targeted single-gene tests, in which specific MMR genes were analyzed, whereas 3 out of 11 patients (27.2%) were identified as harboring PVs or LPVs in an MMR gene by NGS analysis of MMR genes and other genes involved in major hereditary cancer predisposition syndromes. Specifically, as regards the 19 probands with a molecular diagnosis of LS, six different PVs/LPVs in the *MLH1* gene were identified in nine patients (47.4%), four different PVs/LPVs in the *MSH2* gene were identified in four patients (21.1%), two different PVs in the *MSH6* gene were identified in five patients (26.3%), and one PV in the *PMS2* gene was identified in one patient (5.3%) (Table S1, Figure 2). About half of the patients with a molecular diagnosis of LS (10 out of 19, 52.6%) developed more than one LS-related cancer. Considering the probands with a molecular diagnosis of LS (n = 19) together with their mutation carrier relatives with cancer (n = 13), 28 individuals were diagnosed with CRC, with an average age of onset of 45.7 years (range 29–69). The second most frequent cancer was EC, which was diagnosed in eight women, with an average age of onset of 54.9 years (range 48–65) (Table S2). Analysis of the age of cancer onset stratified by each altered MMR gene revealed that the average age of CRC onset was 42.7 years (range 29–63) in the 12 *MLH1* PV/LPV carriers, 38.1 years (range 32–54) in the 8 patients harboring *MSH2* PVs, and 60.8 years (range 45–69) in the 6 patients harboring *MSH6* alterations. An average age of CRC onset was 49.5 years (range 38–61) in two related mutation carriers for *PMS2* gene. For EC, the average age of onset was 53.5 years (range 51–56) in the two carriers of *MLH1* variants, 51.5 years (range 48–55) in the two carriers of *MSH2* variants, and 57.2 years (range 50–65) in the four carriers of *MSH6* variants (Table 2 and Table S2).

**Figure 3:**
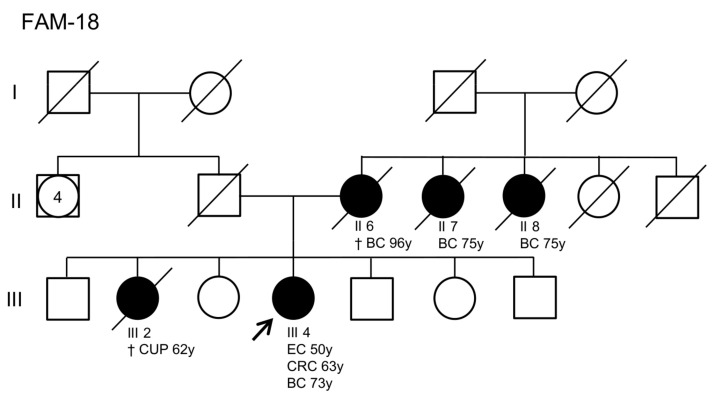
Family pedigree of the patient carrying an exon 1–2 deletion in the *MSH6* gene and a variant of unknown significance (VUS) in the *MSH2* gene. Squares indicate men; circles indicate women. Squares and circles with a number inside represent multiple individuals. The arrow indicates the index case. Black-filled symbols denote individuals with cancer and unfilled symbols indicate individuals without cancer. Slashed symbols denote dead individuals. The following information is given below each filled symbol: clinical manifestations (BC = breast cancer, CRC = colorectal cancer, CUP = cancer of unknown primary, EC = endometrial cancer), age of death (†), age of cancer onset (y = years).

In family 18, a deletion of the first two exons of the *MSH6* gene and the VUS c.728G>A in the *MSH2* gene were identified in the proband by MLPA and NGS analysis, respectively. In addition to developing CRC at the age of 63, this patient was diagnosed with EC and breast cancer at 50 and 73 years, respectively. Based on her family history, one of her sisters died at the age of 62 due to multiple metastases, whose primary tumor is unknown. Furthermore, her mother and two maternal aunts developed breast cancer at over 70 years of age (Figure 3).

### 3.3. Patients with VUS

Among the 30 patients who underwent germline testing, 5 (16.7%) had a VUS for which pathogenicity has not been demonstrated nor excluded in the scientific literature and genetic variant databases. All VUS (n = 6) identified in these patients were detected by NGS (Table S3).

**Figure 4:**
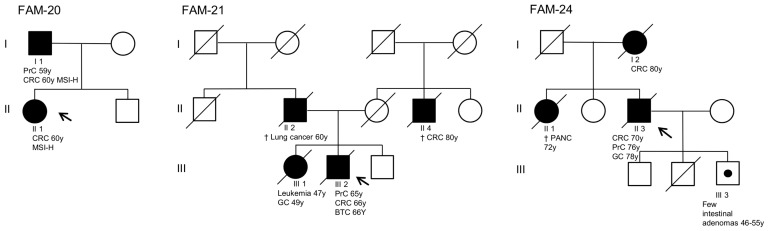
Family pedigrees of patients carrying a variant of unknown significance (VUS) in the *MSH6* (family 20), *PMS2* (family 21), and *APC/BMPR1A* (family 24) genes. Squares indicate men; circles indicate women. The arrow indicates the index case. Black-filled symbols denote individuals with cancer and unfilled symbols indicate individuals without cancer. Symbols enclosing a black dot indicate individuals with intestinal adenomas. Slashed symbols denote dead individuals. The following information is given below each filled symbol: clinical manifestations (BTC = biliary tract cancer, CRC = colorectal cancer, GC = gastric cancer, MSI-H = microsatellite-instability-high, PANC = pancreatic cancer, PrC = prostate cancer), age of death (†), age of cancer onset (y = years).

The variant detected in the *MSH6* gene (c.663A>C; p.Glu222Asn) of the index case of family 20 is reported as a VUS or likely benign variant in the ClinVar database, thus representing a conflict in the submitted interpretations. To better elucidate its role in the patient’s clinical phenotype, we performed a segregation analysis. This variant was not detected in the father, who developed an MSI-H CRC at the age of 60 years, but was found in the unaffected mother (Figure 4). In the index case of family 21, a VUS was identified in the *PMS2* gene (c.184G>A; p.Gly62Ser). The index case was a male patient who developed prostate cancer and CRC at the age of 65 and 66 years, respectively. Based on his family history, his sister was diagnosed with leukemia at 47 years of age and died of gastric cancer at 49 years of age. Moreover, his father died of lung cancer at 60 years of age and his maternal uncle died of CRC at approximately 80 years of age (Figure 4). A probably damaging effect and an alteration in protein function were predicted for this variant by in silico analyses (Table S3). In the index case of family 22, a VUS was identified in the *NBN* gene (c.839C>T; p.Thr280Ile). The index case was a male patient who developed CRC at 58 years of age. A family history of LS-related and non-LS-related cancer was recorded for this individual. In silico analyses indicated that the VUS identified in the *NBN* gene is not expected to affect protein function (Table S3). In the index case of family 23, a VUS was identified in the *ATM* gene (c.3563A>C; p.His1188Pro). The index case was a female patient who developed CRC at 65 years of age, with a positive family history of cancer. In silico analyses predicted that this variant may possibly impair protein function (Table S3). In the index case of family 24, two VUS were identified in the *APC* (c.2780C>G; p.Ala927Gly) and *BMPR1A* (c.1498A>G; p.Met500Val) genes. The index case was a male patient who developed CRC, prostate cancer, and gastric cancer at the age of 70, 76, and 78 years, respectively. As regards his family history, his mother developed CRC at 80 years of age, his sister developed pancreatic cancer at 72 years of age, and his son developed a few intestinal adenomas between 46 and 55 years of age (Figure 4). The VUS in the *APC* gene was predicted to be probably benign, while the VUS in the *BMPR1A* gene was predicted to be disruptive by in silico tools developed to estimate the effects of missense changes on protein function (Table S3).

### 3.4. Patients with Negative Results on Genetic Testing

For 6 of the 30 patients who underwent germline testing, no relevant variants were identified by molecular genetic analysis. In this group, half of the probands underwent NGS analysis and half were subjected to MMR gene analysis. Half of the female probands (2/4) developed EC at an average age of 53 years (range 52–54) (Table S4).

**Figure 5:**
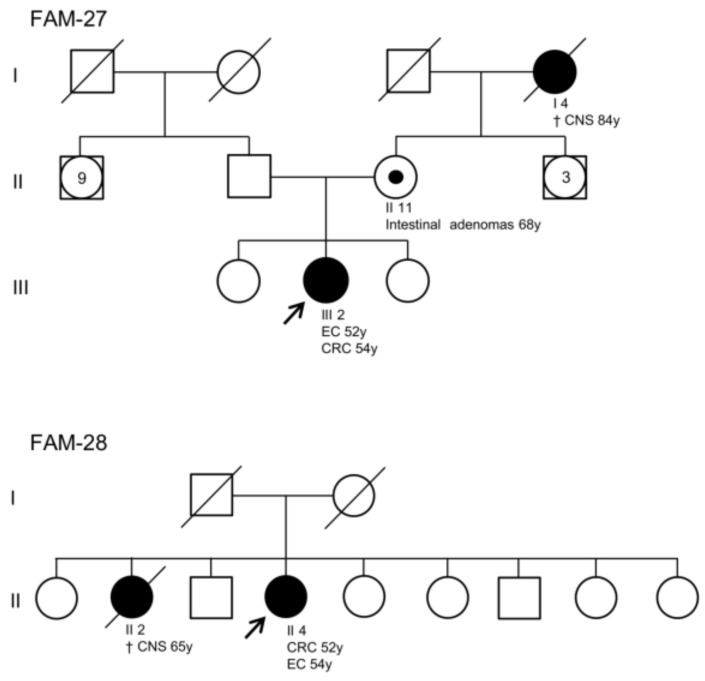
Family pedigrees of patients not carrying germline variants in genes associated with major hereditary cancer predisposition syndromes based on the tests performed in this study. Squares indicate men; circles indicate women. Squares and circles with a number inside represent multiple individuals. The arrow indicates the index case. Black-filled symbols denote individuals with cancer and unfilled symbols indicate individuals without cancer. Symbols enclosing a black dot indicate individuals with intestinal adenomas. Slashed symbols denote dead individuals. The following information is given below each filled symbol: clinical manifestations (CNS = central nervous system cancer, CRC = colorectal cancer, EC = endometrial cancer), age of death (†), age of cancer onset (y = years).

Among the individuals analyzed by NGS, the proband of family 25 developed thyroid cancer at 28 years of age and MSI-H CRC at 46 years of age. Moreover, two of her paternal relatives were diagnosed with thyroid cancer at the age of 40 years (Table S4). The probands of family 27 and family 28 developed CRC and EC at less than 55 years of age (Table S4, Figure 5). However, no variants were identified in the 25 genes analyzed by NGS for these three cases. The probands of family 26, family 29, and family 30 underwent MMR gene analysis and tested negative for alterations in these genes. They did not develop cancers other than CRC and at least one of their relatives developed CRC and/or EC before 50 years of age.

## 4. Discussion

In the presented cohort study, we report the results of tumor and germline mutation testing of 841 CRC patients to identify LS-affected individuals. The selection of suspected LS patients was based on the MSI and *BRAF* molecular status, which were evaluated on CRC specimens in order to discriminate between sporadic and LS-related CRC. Noteworthy, sporadic MSI-H CRC can be associated with other somatic genetic abnormalities, including mutations in the *BRAF* gene, whose V600E variant is the most frequent [20]. Overall, 100 CRCs out of 841 (11.9%) had an MSI-H status, 70 of which (70%) tested negative for the *BRAF*^V600E^ substitution. Of these 70 patients, 30 were examined clinically as part of genetic counseling and subsequently underwent molecular analysis of DNA extracted from peripheral blood. The remaining 40 patients were recommended for clinical evaluation but did not undergo further testing at our institute. Patients at high risk for LS can be identified based on the age of cancer onset and cancer family history in accordance with the Amsterdam and Bethesda criteria [21]. Nevertheless, there are reports of LS not being detected in patients with a personal and/or family history of LS-related cancer fulfilling these clinical criteria [22]. As a result, current practices tend to focus on the evaluation of MMR deficiency in CRC tissues, also, because the results of tumor analysis can influence the therapeutic strategy [23]. In this study, the clinical evaluation of 30 patients with MSI-H and WT for *BRAF*^V600^ CRC identified 19 suspected LS patients with a strong personal and/or family history of LS-related cancer meeting the Amsterdam or Bethesda criteria and 11 suspected LS patients with a personal but not a family cancer history and/or with a family history of cancers potentially correlated to other major hereditary tumor predisposition syndromes. The 19 patients meeting the Amsterdam and Bethesda criteria were subjected to molecular analysis of MMR genes (*MLH1*, *MSH2*, *MSH6*, and *PMS2*) and the *EPCAM* gene, while the remaining 11 patients underwent NGS analysis of 25 genes associated with major hereditary cancer predisposition syndromes, including LS. Through this clinical and molecular approach, we detected a higher proportion of PVs and/or LPVs causative of LS in patients who strictly met LS clinical criteria (16/19, 84.2%) compared to patients having a personal but not a family history of cancer and/or fulfilling the criteria for other major hereditary tumor predisposition syndromes (3/11, 27.2%). These findings indicate that a careful clinical evaluation of personal and family history is crucial for deciding whether or not targeted genetic testing should be performed to establish a molecular diagnosis of LS. Based on the scientific literature, up to 80% of patients with suspected LS carry variants in one of the MMR genes (*MLH1*, *MSH2*, *MSH6*, and *PMS2*) or the *EPCAM* gene [24,25]. In our study, 19 patients out of 30 (63.3%) showed PVs and/or LPVs in a MMR gene. Among these 19 patients, the percentages of individuals with PVs and LPVs in these genes were as follows: 47.4% for *MLH1*, 21.1% for *MSH2*, 26.3% for *MSH6*, and 5.3% for *PMS2*. One of the index cases with a molecular diagnosis of LS (family 18) developed CRC at 63 years of age and EC at 50 years of age, consistent with the reported age of onset of these LS-related malignancies. However, her family’s cancer history revealed the occurrence of non-LS-related tumors in her relatives. MLPA analysis showed that the proband had a germline deletion of *MSH6* exons 1 and 2, which is responsible for the LS phenotype. Furthermore, NGS analysis identified the VUS c.728G>A (p.Arg243Gln) in the *MSH2* gene. Computational prediction with different in silico tools suggested that this variant may have a deleterious impact on protein function. Additionally, it has been observed in LS patients from different families [26,27] and has been described as co-occurring with other pathogenic variants in LS patients [20,22]. Since this evidence is not sufficient to establish a pathogenic role for this variant in LS, further investigations will be needed to elucidate its functional and clinical significance. The *MSH6* c.1975_1960GTGAdup PV was identified as recurrent in our cohort of patients, occurring in four out of five *MSH6*-mutated families (families 14, 15, 16, and 17). This variant results in a premature termination codon that is predicted to cause truncation of the encoded protein or its absence due to nonsense-mediated mRNA decay, which are known mechanisms of disease [28]. Although these families are not currently related to each other, they may have had a common ancestor. Based on the NCCN guidelines, the age of onset of CRC in LS patients is earlier (4th–5th decade of life) compared to the general population [29]. Specifically, the average age of CRC onset is 44 years for *MLH1* and *MSH2* PV/LPV carriers, 42–69 years for *MSH6* PV/LPV carriers, and 61–66 years for *PMS2* PV/LPV carriers. Consistent with these guidelines, the average age of CRC onset in our cohort was 42.7 years (range 29–63) for *MLH1* PV/LPV carriers and 60.8 years (range 45–69) for *MSH6* PV/LPV carriers. On the other hand, the average age of CRC onset in our cohort was 38.1 years (range 32–54) for *MSH2* PV/LPV carriers and 49.5 years (range 38–61) for *PMS2* carriers, which are lower than the average age of CRC onset reported in the NCCN guidelines [29]. 

Furthermore, it is now well established that the cumulative risk of developing cancer varies among LS patients based on specific genetic alterations in MMR genes [30]. More specifically, germline LPVs/PVs in *MLH1* and *MSH2* genes are associated with a higher lifetime CRC risk of 58–82%, while LPVs/PVs in *MSH6* and *PMS2* genes are associated with a lower lifetime CRC risk of 10–22% [30]. 

Much of the clinical heterogeneity observed in LS patients may be explained by the molecular profiles of individual carcinomas, which partly are dependent on which MMR gene is affected [31].

The identification of disease-causing mutations in LS patients guides the clinical management of their entire affected families and has implications for genetic counseling and surveillance. As opposed to PVs and LPVs, VUS do not enable clear establishment of a diagnosis of LS at the molecular level due to their unclear functional implications in disease pathogenesis. As a result, VUS cannot be used to identify asymptomatic relatives. Moreover, carriers of VUS in MMR genes are not followed in surveillance programs, which have proven to reduce morbidity and mortality in LS patients [29]. In our cohort of patients, 5 out of 30 tested patients (16.7%) harbored a VUS. All these patients underwent multigene NGS analysis since they showed a personal and/or family history of cancers potentially correlated to other hereditary tumor predisposition syndromes. Two index cases had a single VUS in one MMR gene (*MSH6* and *PMS2*), one index case had two VUS co-occurring in two genes (*APC* and *BMPR1A*) associated with hereditary colorectal polyposis and juvenile polyposis syndromes, respectively, and the remaining two index cases had a single VUS in other genes (*NBN* and *ATM*) not strictly correlated to hereditary predisposition to CRC. The VUS detected in the *MSH6* gene (c.663A>C; p.Glu221Asp) was identified in a female patient (family 20) who developed CRC at the age of 60 years. This variant is reported in ClinVar with a conflict of interpretation regarding its pathogenetic role in LS clinical manifestations. The segregation analysis performed in the current study showed that this VUS was inherited from the unaffected mother and not from the father who developed CRC. Based on this finding, we hypothesize that it has a benign role in LS. The VUS detected in the *PMS2* gene (c.184G>A; p.Gly62Ser) was identified in a male patient (family 21) who developed prostate cancer, CRC, and biliary tract cancer between 65 and 66 years of age. No LS-related cancers were recorded in his family history. 

To date, this variant has not been reported in the literature in patients with LS and/or other hereditary tumor predisposition syndromes. Nevertheless, in the ClinVar database, two different patients harboring the *PMS2* (c.184G>A; p.Gly62Ser) VUS have been reported in association to LS and other hereditary tumor predisposition syndromes. Computational prediction from different in silico tools suggested that this variant may have a deleterious impact on protein function. Altogether, these findings do not rule out a role for this variant in LS, and further studies are needed to clarify its clinical significance. The two co-occurring VUS (family 24) were detected in genes associated with hereditary colon polyposis syndromes, i.e., *APC* (c.2780C>G; p.Ala927Gly) and *BMPR1A* (c.1498A>G; p.Met500Val) [32,33]. Based on computational protein analysis, the *APC* VUS was predicted to be probably benign, whereas the *BMPR1A* VUS was predicted to have a deleterious effect on protein function. Of note, the son of the index case developed few colon polyps between the age of 46 and 55 years. Moreover, the mother and the sister of the index case developed CRC and pancreatic cancer, respectively, at over 70 years of age. Considering the clinical manifestation of the index case and his relatives, the involvement of these variants in the observed clinical manifestations cannot be excluded. Nevertheless, further clinical and functional studies are needed to elucidate the potential role of these VUS in hereditary colon cancer predisposition syndromes. In our cohort of patients who underwent genetic analysis, 6 out of 30 (20%) were found not to harbor genetic alterations with clinical significance. A total of three index cases were subjected to NGS analysis of 25 genes associated with major hereditary cancer predisposition syndromes based on their clinical findings. The first index case (family 25) was diagnosed with MSI-H CRC at the age of 46. Moreover, he had developed thyroid cancer at the age of 28 and had a family history of thyroid cancer. To date, there are no data indicating an increased incidence of thyroid cancer in LS patients, in contrast with patients carrying mutations in other genes, such as *APC* or *PTEN* [34,35]. The second index case (family 27) was indicated for the same genetic testing, although no cancer familiarity was recorded, because his mother had some colon polyps removed. The third index case (family 28) was suggested for this more extensive analysis (in addition to MMR genes) due to clinical findings of negative cancer family history in addition to the absence of information about both maternal and paternal relatives. The other three index cases were subjected to molecular analysis of MMR genes (*MLH1*, *MSH2*, *MSH6*, and *PMS2*) and the *EPCAM* gene. Specifically, genetic analysis of two of these index cases (family 26 and 29) focused on the MMR genes because they developed MSI-H CRC at less than 50 years of age and had a family member who developed CRC or EC at the age of 56 years. Similarly, the third index case (family 30) underwent genetic analysis for MMR genes because she was diagnosed with MSI-H CRC at 63 years of age and her mother developed CRC at 46 years of age. None of these analyses revealed genetic alterations potentially causative of LS. Overall, 6 out of 30 patients (20%) with a clinical suggestion of LS who were screened for MMR genes remained without a molecular diagnosis. In these patients, further in-depth genetic analysis, such as whole exome sequencing, may allow the detection of currently unknown variants potentially related to LS, providing novel screening strategies for the identification of families at risk of hereditary cancer. Altogether, the results presented in this study may help guide genetic counseling, cancer screening, and risk management in patients with genetic alterations in MMR and other cancer genes.

## 5. Conclusions

In this study, using a comprehensive approach based on tumor testing for the assessment of MSI status, clinical evaluation of patients and their relatives, and genetic analysis, we identified a spectrum of variants in MMR genes and other cancer genes. The clinical and molecular characterization of these patients with MSI-H CRC highlights the importance of personalized medicine to provide tailored genetic counseling, management, and surveillance to families with LS and hereditary cancer.

## Figures and Tables

**Table 1:** Clinicopathological features of patients with high microsatellite instability (MSI-H) and *BRAF*^v600^ wild-type CRC subjected to germline genetic testing for Lynch syndrome (LS).

Probands (n = 30)	
**AGE AT ONSET (years)**	
<50	8
≥50	22
**SEX**	
Male	14
Female	16
**TUMOR TYPE**	
CRC	29
Multiple CRCs	1
**Other associated tumors**	
Gastric cancer	2
Endometrial cancer	8
Ovarian cancer	2
Prostate cancer	2
Biliary tract cancer	1
Other cancers	6
**FAMILY HISTORY OF CANCER**	29
CRC	1
Non-CRC	7
CRC and non-CRC	21
**NO FAMILY HISTORY OF CANCER**	1
**GENETIC TESTING RESULTS FOR LS**	
***LPV/PV***	
*MLH1*	9
*MSH2*	4
*MSH6*	5
*PMS2*	1
*EPCAM*	0
***VUS***	
*MLH1*	0
*MSH2*	1
*MSH6*	1
*PMS2*	1
*EPCAM*	0
Other genes: *APC*, *ATM*, *BMPR1A*, *NBN*	4
***Negative Results***	6

Abbreviations: CRC: colorectal cancer; LPV: likely pathogenic variant; PV: pathogenic variant; VUS: variant of unknown significance; Negative Results: No LPV/PV identified in the genes analyzed.

**Table 2:** Average age of CRC and EC onset stratified by single altered mismatch repair gene in patients with high microsatellite instability (MSI-H) and *BRAF*^V600^ wild-type CRC.

Gene	CRC			EC		
	n	Average Age (y)	Range (y)	n	Average Age (y)	Range (y)
*MLH1*	12	42.7	29–63	2	53.5	51–56
*MSH2*	8	38.1	32–54	2	51.5	48–55
*MSH6*	6	60.8	45–69	4	57.2	50–65
*PMS2*	2	49.5	38–61	-	-	-

Abbreviations: CRC: colorectal cancer; EC: endometrial cancer, n: number of patients; y: years.

## Data Availability

The data presented in this study are available in this article.

## Supplementary Materials

The following supporting information can be downloaded at: https://www.mdpi.com/article/10.3390/cancers15205061/s1, Table S1: Main clinicopathological characteristics of 30 MSI-H and *BRAF*^V600^ WT CRC; Table S2: Clinical and molecular features of patients with high microsatellite instability (MSI-H) and *BRAF*^v600^ wild-type CRC carrying pathogenic variants in mismatch repair (MMR) genes; Table S3: Clinical and molecular features of patients with high microsatellite instability (MSI-H) and *BRAF*^V600^ wild-type CRC carrying variants of unknown significance; Table S4: Clinical features of patients with negative results on genetic testing.
