# Supplementary material for: Tumor Testing and Genetic Analysis to Identify Lynch Syndrome Patients in an Italian Colorectal Cancer Cohort
Source: Cancers (Basel). 2023 Oct 19;15(20):5061. doi: 10.3390/cancers15205061 (PMC10605602; doi:10.3390/cancers15205061)
Supplement: Supplementary file 1 [file cancers-15-05061-s001.zip › Supplementary_Table_S1.pdf]

Supplementary Table S1. Main clinicopathological characteristics of 30 MSI-H and *BRAF*^{V600} Wild-type CRC.

	Sex	Age of CRC onset (years)	Location of Tumor	Histological features	TNM Stage*	AJCC Stage*
FAM-1	Male	52	Right Sided	Well differentiated adenocarcinoma	T3N0M0	IIa
FAM-2	Male	40	Right Sided	Mucinous adenocarcinoma, poorly differentiated	T4bN0M0	IIc
FAM-3	Female	46	Right Sided	Mucinous adenocarcinoma, poorly differentiated	T3N0M0	IIa
FAM-4	Male	37	Left Sided	Mucinous adenocarcinoma, moderately differentiated	T4aN2M0	IIIc
FAM-5	Male	29	Right Sided	Undifferentiated adenocarcinoma	T4bN2aM0	IIIc
FAM-6	Male	50	Transverse	Moderately differentiated adenocarcinoma	T4aN0M0	IIb
FAM-7	Female	49	Right Sided	Poorly differentiated adenocarcinoma	T4aN0M0	IIb
FAM-8	Female	63	Right Sided	Mucinous adenocarcinoma, poorly differentiated	T4aN0M0	IIb
FAM-9	Female	36	Right Sided	Mucinous adenocarcinoma, moderately differentiated	T4N2M0	IIIc
FAM-10	Male	35	Right Sided	Mucinous adenocarcinoma, poorly differentiated	T4aN0M0	IIb
FAM-11	Female	32	Right Sided	Mucinous adenocarcinoma	T2N1M0	IIIa
FAM-12	Male	38	Sigma	Well differentiated adenocarcinoma	T3N0M0	IIa
FAM-13	Female	54	Right Sided	Mucinous adenocarcinoma, moderately differentiated	T4bN0M0	IIc
FAM-14	Female	66	Transverse	Mucinous adenocarcinoma	T4aN0M0	IIb
FAM-15	Female	60	Left Sided	Poorly differentiated adenocarcinoma	T4bN0M1a	IVa
FAM-16	Female	69	Right Sided	Mucinous adenocarcinoma, poorly differentiated	T3N0M0	IIa
FAM-17	Male	62	Right Sided	Mucinous adenocarcinoma, poorly differentiated	T3N0M0	IIa
FAM-18	Female	63	Left Sided	Mucinous adenocarcinoma, well differentiated	T4bN1M0	IIIc
FAM-19	Male	38	Right Sided	Well differentiated adenocarcinoma	T1N0M0	I
FAM-20	Female	60	Right Sided	Moderately differentiated adenocarcinoma	T4aN0M0	IIb
FAM-21	Male	66	Right Sided	Mucinous adenocarcinoma, moderately differentiated	T4aN2bM1c	IVc
FAM-22	Male	58	Right Sided	Moderately differentiated adenocarcinoma	T4aN2bM1b	IVb
FAM-23	Female	65	Right Sided	Moderately differentiated adenocarcinoma	T2N0M0	I
FAM-24	Male	70	Right Sided	Poorly differentiated adenocarcinoma	T3N0M0	IIa
FAM-25	Female	46	Right Sided	Moderately differentiated adenocarcinoma	T2N0M0	I
FAM-26	Male	47	Right Sided	Mucinous adenocarcinoma	T4aN0M0	IIb
FAM-27	Female	54	Transverse	Mucinous adenocarcinoma	T3N0M0	IIa
FAM-28	Female	52	Left Sided	Poorly differentiated adenocarcinoma	T3N2M0	IIIc
FAM-29	Male	50	Left Sided	Moderately differentiated adenocarcinoma	T3N0M0	IIa
FAM-30	Female	63	Right Sided	Mucinous adenocarcinoma, moderately differentiated	T1N0M0	I

*American Joint Committee on Cancer (AJCC) TNM Staging Classification for Colon Cancer 8th ed., 2017
Abbreviations: CRC: colorectal cancer; MSI-H: high microsatellite instability.
