# Supplementary material for: Tumor Testing and Genetic Analysis to Identify Lynch Syndrome Patients in an Italian Colorectal Cancer Cohort
Source: Cancers (Basel). 2023 Oct 19;15(20):5061. doi: 10.3390/cancers15205061 (PMC10605602; doi:10.3390/cancers15205061)
Supplement: Supplementary file 1 [file cancers-15-05061-s001.zip › Supplementary_Table_S2.pdf]

Supplementary Table S2. Clinical and molecular features of patients with high microsatellite instability (MSI-H) and *BRAF*[v600] wild-type CRC carrying pathogenic variants in mismatch repair (MMR) genes.

	PROBAND									FAMILY HISTORY		
	Age at enrollment (years)	Sex	Gene[a,b,c]	HGVS nomenclature	Protein change	ClinVar assertion	Age of CRC onset (years)	LS-related cancer (age of onset, years)	Any cancer (age of onset, years)	Mutation carrier with cancer (age of onset, years)	Mutation carrier without cancer (age of onset or age at genetic test, years)	Not tested, with cancer (age of onset, years)
**FAM-1**	52	M	*MLH1*[a]	c.380+2T>C	splicing	LPV	52			maternal uncle, CNS (50), ureteral cancer (65); maternal cousin CRC (47)	mother, endometrial polyps (58),; brother (60)	
**FAM-2**	68	M	*MLH1*[a]	c.545+3A>G	splicing	PV	40		Lipomas (63)	sister*, CRC (31)	Nephew (42)	brother, CRC (51)
**FAM-3**	48	F	*MLH1*[a]	c.683dupT	p.Ile229fs	PV	46	OvC (31)	Thymoma (46)			sister, BC (39) ; Mother, BC (42) ; maternal uncle, PrC (73); maternal uncle, CRC (70); maternal aunt, BC (50), EC (51), CRC (53)
**FAM-4**	37	M	*MLH1*[a]	c.731G>A	p.Gly244Asp	PV	37				mother (60)	maternal grandfather, CRC (60†), GC (60†)
**FAM-5**	45	M	*MLH1*[a]	c.731G>A	p.Gly244Asp	PV	29, 44					mother, KC (54†); maternal aunt CRC (65†)
**FAM-6**	50	M	*MLH1*[b]	c.1036C>T	p.Gln346Ter	PV	50			mother, EC (56)		maternal aunt, bilateral BC (40),; maternal grandmother, CRC (77†)
**FAM-7**	49	F	*MLH1*[a]	c.1961C>T	p.Pro654Leu	PV	49	OvC (49)		uncle, CRC (32)	daughter (27); cousin (38)	father, CRC (46†); paternal uncle, CRC (65); grandfather, GC (56†)
**FAM-8**	63	F	*MLH1*[a]	c.1961C>T	p.Pro654Leu	PV	63	EC (51)	Melanoma (51)			Father, PrC (70); Mother, BTC (66); maternal uncle, GC (75); maternal cousin, BC (55); maternal cousin, BC (n.d.); maternal cousin, GC (n.d.)
**FAM-9**	37	F	*MLH1*[a]	c.1961C>T	p.Pro654Leu	PV	36				mother (57); brother (27); sister (34)	
**FAM-10**	35	M	*MSH2*[a]	c.943-1G>A	splicing	PV	35				mother, endometrial polyps (55); sister (40)	maternal aunt, BC (73)
**FAM-11**	32	F	*MSH2*[a]	c.1681G>T	p.Glu561Ter	PV	32			father*, CRC (33†); paternal uncle, CRC (35); paternal cousin, CRC (35)		paternal uncle, CRC (50); paternal uncle, BlC (70); paternal cousin, OvC (40)

**FAM-12**	61	M	*MSH2*[a]	c.1786_1788 delAAT	p.Asn596del	PV	38			nephew, CRC (43)	son (30);<br>brother (70)	
**FAM-13**	54	F	*MSH2*[a]	c.2635-2A>G	splicing	LPV	54	EC (55)		sister, EC (48)	brother (46);<br>son (22)	mother, CRC (56)
**FAM-14**	70	F	*MSH6*[a]	c.1957_1960 GTGAdup	p.Met654fs	PV	66	EC (65)		daughter, BC (41)	sister (50); daughter (48)	mother, CRC (78);<br>brother, benign CNS tumor (40)
**FAM-15**	61	F	*MSH6*[a]	c.1957_1960 GTGAdup	p.Met654fs	PV	60	EC (50)				father, CRC (70);<br>paternal aunt, BC (60†);<br>paternal aunt, BC (50)
**FAM-16**	69	F	*MSH6*[a]	c.1957_1960 GTGAdup	p.Met654fs	PV	69	EC (64)	Lung Cancer (66)	daughter, CRC (45);	son (48);<br>daughter (42);<br>sister (n.d.)	sister, CRC (46), BC (50)
**FAM-17**	62	M	*MSH6*[b]	c.1957_1960 GTGAdup	p.Met654fs	PV	62	GC (44)				father, Laryngeal Tumor (72);<br>paternal uncle, CRC (67†);<br>maternal uncle, CNS (68†);<br>maternal cousin, CRC (60);<br>maternal grandmother, EC (55)
**FAM-18**	73	F	*MSH6*[b]<br><br>*MSH2*[b]	Deletion exon 1-2<br>c.728G>A	 <br>p.Arg243Gln	PV<br><br>VUS	63	EC (50)<br>BC (73)				mother, BC (96†);<br>maternal aunt, BC (75);<br>maternal aunt, BC (75)
**FAM-19**	55	M	*PMS2*[a]	c.1987G>T	p.Glu663Ter	PV	38			Mother, CRC (61)	Niece (26);<br>sister (51);<br>daughter (23);<br>sister (54)	sister, sarcoma (52†);<br>maternal uncle, CRC (75†)

a. Patient analyzed by sequencing and copy number variation of MMR genes
b. Patient analyzed for 25 genes associated with major hereditary cancer predisposition syndromes and copy number variation of MMR genes
Variant positions are based on the following reference transcripts: NM_000249.4 (*MLH1*); NM_000251.3 (*MSH2*); NM_000179.3 (*MSH6*); NM_000535.7 (*PMS2*).
Abbreviations: †: death; *: obligate carrier; BC: breast cancer; BlC: bladder cancer; BTC: biliary tract cancer; CRC: colorectal cancer; CNS: central nervous system cancer; EC: endometrial cancer; FAM: family; GC: gastric cancer; HGVS: human genome variation society; KC: kidney cancer; LPV: likely pathogenic variant; n.d.: not determined; OvC: ovarian cancer; PANC: pancreatic cancer; PrC: prostate cancer; PV: pathogenic variant; VUS: variant of unknown significance.
