# Supplementary material for: Tumor Testing and Genetic Analysis to Identify Lynch Syndrome Patients in an Italian Colorectal Cancer Cohort
Source: Cancers (Basel). 2023 Oct 19;15(20):5061. doi: 10.3390/cancers15205061 (PMC10605602; doi:10.3390/cancers15205061)
Supplement: Supplementary file 1 [file cancers-15-05061-s001.zip › Supplementary_Table_S3.pdf]

Supplementary Table S3. Clinical and molecular features of patients with high microsatellite instability (MSI-H) and *BRAF*^{V600} wild-type CRC carrying variants of unknown significance.

PROBAND													FAMILY HISTORY (age of onset or age at genetic test, years)
	Age at enrollment (years)	Sex	Gene	HGVS nomenclature	Clinvar assertion	Protein change	Panther Db prediction	Polyphen-2 prediction	Sift prediction	Age of CRC (age of onset, years)	LS-related cancer (age of onset, years)	Any cancer (age of onset, years)	
**FAM-20**	65	F	*MSH6*	c.663A>C	VUS/LB	p.Glu221Asp	Possibly damaging (Pdel=0.5)	Benign (score=0.002)	Tolerated (score 0.26)	60			Father, PrC (59), CRC (60); mother unaffected carrier (88)
**FAM-21**	66	M	*PMS2*	c.184G>A	VUS	p.Gly62Ser	Probably damaging (Pdel=0.95)	Probably damaging (score=1.00)	Affect protein function (score=0.01)	66	PrC (65), BTC (66)		Sister, leukemia (47), GC (49†); father lung cancer (60†); uncle CRC (80†),
**FAM-22**	59	M	*NBN*	c.839C>T	VUS	p.Thr280Ile	Probably benign (Pdel=0.27)	Benign (score=0.000)	Tolerated (score=0.68)	58			sister PANC (51); father CRC (75); mother, BC (60), PANC (81); maternal cousin, GC (62†)
**FAM-23**	66	F	*ATM*	c.3563A>C	VUS	p.His1188Pro	n.a.	Possibly damaging (score=0.666)	Affect protein function (score=0.01)	65			father, BTC (74); mother, CRC (67); maternal uncle, CRC (70†)
**FAM-24**	78	M	*APC*	c.2780C>G	VUS	p.Ala927Gly;	Probably benign (Pdel=0.19)	n.a.	Tolerated (score=0.08)	70	GC (77); PrC (76)	epithelioma (n.d.)	sister, PANC (72†); son, intestinal adenomas (46-55); mother, CRC (80).
			BMPR1A	c.1498A>G	VUS	p.Met500Val	Probably benign (Pdel=0.13)	Probably damaging (score=1.00)	Affect protein function (score=0.02)				

Variant positions are based on the following reference transcripts NM_000179.3 (*MSH6*); NM_000535.7 (*PMS2*); NM_002485.5 (*NBN*); NM_000051.4 (*ATM*); NM_000038.6 (*APC*); NM_004329.3 (*BMPR1A*);

Abbreviations: †: death; BC: breast cancer; BTC: biliary tract cancer; CRC: colorectal cancer; EC: endometrial cancer; FAM: family; GC: gastric cancer; HGVS: human genome variation society; LB: likely benign variant; ; n.a.: not available; n.d.: not determined; PANC: pancreatic cancer; Pdel: probability of deleterious effect (Pantherdb, https://www.pantherdb.org/); PrC: prostate cancer; VUS: variant of unknown significance.
