# Supplementary material for: Tumor Testing and Genetic Analysis to Identify Lynch Syndrome Patients in an Italian Colorectal Cancer Cohort
Source: Cancers (Basel). 2023 Oct 19;15(20):5061. doi: 10.3390/cancers15205061 (PMC10605602; doi:10.3390/cancers15205061)
Supplement: Supplementary file 1 [file cancers-15-05061-s001.zip › Supplementary_Table_S4.pdf]

Supplementary Table S4. Clinical features of patients with negative results on genetic testing.

PROBAND						FAMILY HISTORY (age of onset, years)
	**Age at enrollment (years)**	**Sex**	**Age of CRC onset (years)**	**LS-related cancer (age of onset, years)**	**Any cancer (age of onset, years)**	
**FAM-25[b]**	47	F	46		THCA (28)	paternal cousin, THCA (40);<br>paternal cousin, THCA (40);<br>paternal uncle, lung Cancer (80†)
**FAM-26[a]**	47	M	47			father, lung Cancer (65†);<br>paternal uncle, CRC (56)
**FAM-27[b]**	55	F	54	EC (52)		maternal grandmother, CNS (84†);<br>mother, intestinal adenomas (68y)
**FAM-28[b]**	54	F	52	EC (54)		sister, CNS (65†)
**FAM-29[a]**	50	M	50			mother, EC (56)
**FAM-30[a]**	63	F	63			mother, CRC (46);<br>paternal aunt, CRC (68);<br>paternal cousin, BC (55)

a. Patient with high microsatellite instability (MSI-H) and $BRAF^{V600}$ wild-type CRC analyzed by sequencing and copy number variation of MMR genes
b. Patient with high microsatellite instability (MSI-H) and $BRAF^{V600}$ wild-type CRC analyzed for 25 genes associated with major hereditary cancer predisposition syndromes.
Abbreviations: †: death; BC: breast cancer; CNS: central nervous system cancer; CRC: colorectal cancer; EC: endometrial cancer; THCA: thyroid cancer.
